# Lentiviral Vectors Expressing Chimeric NEDD4 Ubiquitin Ligases: An Innovative Approach for Interfering with Alpha-Synuclein Accumulation

**DOI:** 10.3390/cells10113256

**Published:** 2021-11-21

**Authors:** Stefania Vogiatzis, Michele Celestino, Marta Trevisan, Gloria Magro, Claudia Del Vecchio, Deran Erdengiz, Giorgio Palù, Cristina Parolin, Kathleen Maguire-Zeiss, Arianna Calistri

**Affiliations:** 1 Department of Molecular Medicine, University of Padua, Via A. Gabelli 63, 35121 Padua, Italy; stefi.voy@gmail.com (S.V.); michele.celestino.uni@gmail.com (M.C.); marta.trevisan@unipd.it (M.T.); gloria.magro@phd.unipd.it (G.M.); claudia.delvecchio@unipd.it (C.D.V.); giorgio.palu@unipd.it (G.P.); cristina.parolin@unipd.it (C.P.); 2 Department of Neuroscience, Georgetown University Medical Center, 3970 Reservoir Rd NW, NRB, EP04, Washington, DC 20057, USA; de206@georgetown.edu (D.E.); Kathleen.MaguireZeiss@georgetown.edu (K.M.-Z.)

**Keywords:** NEDD4, ubiquibodies, alpha-synuclein, Parkinson’s disease

## Abstract

One of the main pathological features of Parkinson’s disease (PD) is a diffuse accumulation of alpha-synuclein (aS) aggregates in neurons. The NEDD4 E3 Ub ligase promotes aS degradation by the endosomal–lysosomal route. Interestingly, NEDD4, as well as being a small molecule able to trigger its functions, is protective against human aS toxicity in evolutionary distant models. While pharmacological activation of E3 enzymes is not easy to achieve, their flexibility and the lack of “*consensus*” motifs for Ub-conjugation allow the development of engineered Ub-ligases, able to target proteins of interest. We developed lentiviral vectors, encoding well-characterized anti-human aS scFvs fused in frame to the NEDD4 catalytic domain (ubiquibodies), in order to target ubiquitinate aS. We demonstrate that, while all generated ubiquibodies bind to and ubiquitinate aS, the one directed against the non-amyloid component (NAC) of aS (Nac32HECT) affects aS’s intracellular levels. Furthermore, Nac32HECT expression partially rescues aS’s overexpression or mutation toxicity in neural stem cells. Overall, our data suggest that ubiquibodies, and Nac32HECT in particular, represent a valid platform for interfering with the effects of aS’s accumulation and aggregation in neurons.

## 1. Introduction

Parkinson Disease (PD) is the second most frequent neurodegenerative disease after Alzheimer’s, affecting more than 1% of people over 55 years old and more than 3% of people over 75 years old [1]. PD is characterized by the loss of dopaminergic neurons in the *substantia nigra pars compacta*, and the subsequent striatal dopamine deficiency, which primarily manifests as a motor disorder. In 80% of patients, PD involves non-motor symptoms, including dementia [2]. PD is also defined as a complex multifactorial disease, resulting from the combination of environmental and genetic factors, both of which play a decisive role in the development of the disease. In particular, different toxic elements present in the environment (e.g., pesticides, chemical agents, pollution, or viruses) have been explored as triggers or co-factors of PD pathogenesis, acting either at the olfactory bulb or at the gut level, before spreading to the central nervous system (CNS) via olfactory or vagus nerves [3].

The degeneration of the *substantia nigra pars compacta* and the presence of Lewy’s bodies (LBs), which represent anomalous cytoplasmic proteinaceous inclusions, mainly consisting of the alpha-synuclein protein (aS), are considered the hallmark of the disease [4]. While the role of LBs in the pathogenesis of PD is still controversial [4] and their identification as deposits of misfolded aS has led to the classification of PD as a synucleinopathy [5]. Alpha-synuclein, encoded by the *SNCA* gene, is a multifunctional natively unfolded protein under physiological conditions that functions by interacting with cellular membranes [6,7]. Strong evidence suggest that aS exploits its unfolded structure to acquire alternative ordered conformations, enabling the protein to interfere with different cellular processes within the CNS [6]. However, this plasticity can also lead to aS self-binding through its non-amyloid component (NAC) domain, resulting in a stable β-sheet structure. This happens, for instance, when aS intracellular levels raise above a certain threshold, or in the presence of specific NAC mutations.

Indeed, aS was the first protein implicated in familial PD. Approximately 5–10% of PD are monogenic familial forms with either an autosomal dominant or a recessive inheritance pattern [8]. A53T aS missense mutation is associated with autosomal dominant early-onset PD [9,10]. Not only specific mutations in *SNCA*, but also its presence in extra wild-type (WT) copies, are associated with PD, with an apparent dosage effect [11,12]. Both mutations and multiplication of *SNCA* are likely to promote aS misfolding or aggregation that, in sporadic PD, might be induced by still undefined genetic or epigenetic factors (or both) [13]. Accordingly, with a role for unfolded aS or protein accumulation, defects in protein degradation pathways were also associated to familial PD cases. Most cellular proteins are targeted for degradation by conjugation with ubiquitin (Ub) chains. Protein ubiquitination involves an enzymatic cascade, starting with the Ub-activating enzyme/E1, followed by the Ub-conjugating enzyme/E2 and by the Ub-ligase/E3, which form an isopeptide bond between the carboxyl terminus of Ub and the alpha-amino group of a lysine residue on the target protein. Mutations in Parkin, a protein with E3 activity, and in the Ub C-terminal hydrolase L1, suggest that enzymes in the Ub pathway could provide a molecular link between the accumulation of aS with defects in protein degradation systems [14]. It has been demonstrated that while Parkin can ubiquitinate a glycosylated form of aS [15], the neuronally expressed, developmentally down-regulated gene 4 (NEDD4) Ub-ligase conjugates K63-Ub chains to aS and targets it for degradation through the lysosomes. In agreement, a down-regulation of NEDD4 levels increases aS contents, a process that requires components of the endosomal sorting complex required for transport (ESCRT) machinery [16]. NEDD4 has a modular structure consisting of an N-terminal C2 domain that determines the specificity of binding to the substrate, four WW domains involved in protein–protein interactions, and a homologous to E6-AP carboxyl terminus (HECT) catalytic domain [16]. Importantly, Tofaris and colleagues convincingly showed that NEDD4 is overexpressed in the brain regions presenting LB, and that a single nucleotide polymorphism in its coding sequence is associated with a greater risk of PD onset [17]. Overall, this work correlated NEDD4 with aS turnover and PD. Furthermore, NEDD4 is protective against human aS toxicity in evolutionary distant models [18]. In this context, a small molecule, able to bind to and activate NEDD4 (NAB2) functions, was identified as neuroprotective agent in neuronal models of aS toxicity [19]. However, since E3 activation cannot be easily obtained pharmacologically and despite promising results with compound screens [19], data supporting this strategy are still limited. E3s are usually the main determinants of substrate specificity in the Ub proteolytic pathways. Their conformational flexibility, along with the fact that “*consensus*” motifs are not required for Ub conjugation, support the possibility of engineering Ub-ligases to specifically target proteins of interest. In this context, chimeras have been generated, called “ubiquibodies”, that combine the catalytic activity of E3 Ub-ligases with single chain variable fragments (scFvs), which can be used to target virtually any protein for degradation, while losing their ability to modify their natural target [20]. Furthermore, work carried out to clarify the cell machinery involved in human immunodeficiency virus (HIV) budding has previously shown that the ubiquitination activity of NEDD4 can be directed towards proteins of interest by fusing its HECT domain to heterologous factors, binding that selected target [21]. 

Here, we developed chimeric NEDD4 Ub-ligases specifically targeting aS, as a tool for interfering with aS accumulation. In particular, we generated chimeras constituted by the HECT catalytic domain of NEDD4, fused in frame to different scFvs, specifically recognizing aS. To this end, three previously characterized scFvs were selected, as follows: Nac32—directed against the NAC domain of human aS [22]; d5e—which exclusively binds the oligomeric form of aS [23]; and d10—interacting with both monomeric and oligomeric forms of the protein (panspecific) [24]. Developed ubiquibodies were cloned into a third-generation, self-inactivating lentiviral vector. We were able to show that recombinant lentiviral particles efficiently transduce human embryonic kidney 293T cells, mouse dopaminergic neuroblastoma cells (MN9D cells), and neural stem cells (NSCs) derived from PD patients. Furthermore, the ubiquibody, based on Nac32 (Nac32HECT), interacts with and ubiquitinates aS, as well as reducing aS by an intracellular amount. Importantly, Nac32HECT expression partially reverses toxic effects of aS’s overexpression or mutation in NSCs. The results achieved so far represent a starting point strongly supporting the validity of this strategy to obtain a specific degradation of aS.

## 2. Materials and Methods

### 2.1. Cell Lines and Cells Derived from Induced Pluripotent Stem Cells

Human embryonic kidney 293T cells (ATCC^®^ CRL-3216™) and the neuroblastoma derived SH-SY5Y cell line (ATCC^®^ CRL-2266) were grown in Dulbecco’s modified Eagle’s medium (Gibco™, Life Technologies™, Woburn, MA, United States), with the addition of 10% (*v*/*v*) heat-inactivated foetal bovine serum (Gibco™, Life Technologies™, Woburn, MA, United States). 

MN9DwtsynIRESgfp (MN9Dsyn) cells derive from the MN9D dopaminergic-like cell line that was obtained from mouse embryonic mesencephalon [25]. MN9Dsyn cells express human wild type (WT) aS, along with the green fluorescent protein (GFP), under the transcriptional control of a doxycycline inducible promoter [26]. MN9Dsyn cells were grown in DMEM supplemented with 5% *v*/*v* heat-inactivated FBS and 200 μg/mL hygromycin B (ThermoFisher Scientific, Woburn, MA, USA).

UNIPDi007-A human-induced pluripotent stem cell (hiPSC) lines were derived from human fibroblast BJ cell (ATCC^®^ CRL-2522™), reprogrammed by Yamanaka factors, as previously described [27]. Two iPSC lines originating from PD patients (ND50040 and ND50050, respectively) were purchased from RUCDR Infinite biologics^®^ (Piscataway NJ, USA). These cells display the following features: ND50040 bears *SNCA* triplication, while ND50050 is characterized by A53T aS. Human iPSCs were seeded on Geltrex™-coated plates and were grown in mTeSR medium (Stem Cell, Voden, Meda (MI), Italy). 

Neural stem cells (NSCs) were obtained by culturing hiPSCs on Geltrex™-coated dishes with PSC Neural Induction Medium (Gibco™, Life Technologies™, Woburn, MA, USA). To differentiate NSCs into dopaminergic progenitors (DPs), NSCs were grown at high confluency (70%) for 7 days on Poly-ornithine/laminin (Sigma-Aldrich, St. Louis, MO, USA)-coated dishes in DMEM/F12, with 1% *v*/*v* of N2 supplement (Life Technologies™, Woburn, MA, USA), 200 ng/mL of Sonic Hedgehog (SHH; Provitro, Berlin, Germany), and 100 ng/mL of Fibroblast Growth Factor 8 (FGF8; Provitro). After 7 days, DPs were plated on Poly-ornithine/laminin coated dishes, in DMEM/F12, 1% of N2 supplement, 20 ng/mL of brain-derived neurotrophic factor (BDNF; Stem Cell, Voden), 0.2 mM of ascorbic acid (AA; Sigma-Aldrich), 20 ng/mL of Glial cell-derived neurotrophic factor (GDNF; ORF genetics, Voden), 0.5 mM of N6,2′-O-Dibutyryladenosine 3′,5′-cyclic monophosphate (dbcAMP; Sigma-Aldrich), and 1 ng/mL of Transforming Growth Factor beta3 (TGFb3; Provitro). At day 7, the medium was swapped in Neurobasal medium and supplemented with 2% *v*/*v* of B27 (Life Technologies™), 1% *v*/*v* of N2 and BDNF, AA, GDNF, dbcAMP, and TGFb3 (concentrations same as above). 

### 2.2. Generation of Lentiviral Vectors Expressing Ubiquibodies and aS

The third generation self-inactivating HIV-derived lentiviral vector pRRLsin18.cPPT.hCMV.GFP.WPRE, herein named pptCMVEGFPWPRE (kindly provided by L. Naldini, Università Vita e Salute San Raffaele, Milan, Italy), expressing the enhanced GFP (EGFP) gene under the transcriptional control of the human cytomegalovirus (HCMV) promoter, was used to clone ubiquibodies and their respective controls.

The NEDD4 sequence (NCBI Reference Sequence: NM_006154.3) was obtained by BamHI and SalI restriction of the pBJ5-NEDD4-WT/C876S plasmids, kindly provided by H. Göttlinger (Massachusetts University, USA), and then cloned into the pptCMVEGFPWPRE in place of the reporter EGFP gene, excised with the same restriction enzymes. The following vectors were developed: pptCMV-NEDD4HA-WT-WPRE and pptCMV-NEDD4HA-C/S-WPRE.

To generate ubiquibodies, synthetic sequences (BioFab, Rome, Italy) encoding for the appropriate scFv (Nac32, d10, d5e) fused in frame to a GSGSG linker, followed by the HECT catalytic domain of the NEDD4 Ub ligase, respectively, in the WT or C867S (C/S) mutated form and enriched with an HA epitope at the 3′ end, were inserted within the pptCMVEGFPWPRE lentiviral vector in place of the EGFP gene. The following vectors were developed: pptCMV-Nac32HECTHA-WT-WPRE, pptCMV-Nac32HECTHA-C/S-WPRE, pptCMV-d10HECTHA-WT-WPRE pptCMV-d10HECTHA-C/S-WPRE, pptCMV-d5eHECTHA-WT-WPRE, and pptCMV-d5eHECTHA-C/S-WPRE. 

To generate pptCMVasynWT3′FWPRE, the plasmid pHM6-alphasynuclein-WT that contains the sequence encoding for WT aS (NCBI Reference Sequence: NM_000345.3) was adopted. pHM6-alphasynuclein-WT was a gift from David Rubinsztein (Addgene plasmid # 40824; http://n2t.net/addgene:40824 (accessed on 1 November 2021)). Coding region was PCR, amplified and enriched with a flag tag, and with a NotI site at the 3′ end. The PCR product was cloned into the pCR 2.1-TOPO vector (Invitrogen), following the manufacturers’ instructions. Next, the fragment excised by HindIII and NotI restriction was cloned into the pptCMVEGFPWPRE lentiviral vector, in place of the EGFP encoding sequence.

To generate the pptCMVEGFPasynWTWPRE lentiviral vectors, the sequence encoding WT aS, fused in frame to the EGFP (GFP-aS WT), was excised by restriction enzymes (HindIII/EcoRI) from the plasmid EGFP-alphasynuclein-WT and cloned into the pptCMVEGFPWPRE lentiviral vector, opened up with the same restriction enzymes. EGFP-alphasynuclein-WT was a gift from David Rubinsztein (Addgene plasmid # 40822; http://n2t.net/addgene:40822 (accessed on 1 November 2021)).

All recombinant plasmids were verified by enzymatic restriction and correct clones were further confirmed by Sanger sequencing.

### 2.3. Production and Titration of Recombinant Lentiviral Particles 

The generated lentiviral vector DNAs were co-transfected along with the packaging plasmids pMDL, pVSV-G, and pRSV-Rev (a kind gift of L. Naldini, Università Vita e Salute San Raffaele, Milan, Italy) in 293T cells, by adopting calcium phosphate reagents, as previously described [28]. Forty-eight hours post-transfection, cell supernatants containing the recombinant lentiviral particles (RLPs) were harvested, filtered with a 0.45-μm-pore-size membrane (Millipore, Bedford, MA, USA), and concentrated 100 times by ultracentrifugation (27,000 rpm, 2 h, 4 °C in a Beckman SW28 rotor), or by adopting VivaSpin columns (Sartorius, Goettingen, Germany). RLPs expressing GFP-aS WT were titrated by flow cytometry analysis (FACSCalibur, Beckton & Dickinson, Franklin Lakes, NJ, USA), upon transduction of 293T cells with serial dilutions [29]. RLPs expressing ubiquibodies were titrated by adopting the HIV-1 p24 ELISA kit (XpressBio, XB-1000), following manufacturer’s instructions. Briefly, 4 μL of concentrated supernatant were serially diluted and dilutions 1:50,000 and 1:100,000 were loaded onto an ELISA plate. Upon adding the lysis reagent, samples were incubated over night at room temperature. Next, the substrate solution was added and 10 min later, stop solution was put in each well and the absorbance was read at the spectrophotometer (450 nm). A standard curve was generated, accepting R^2^ values ≥ 0.95, and the transducing units per ml (TU/mL) were calculated by adopting the following formula:

$$Titer\;\left(TU/ml\right)=\frac{A450-b}{m}\times 100\times dilution\;factor$$

where *b* and m are the coefficients of the standard curve (*y = mx+ b*).

### 2.4. Transduction of Different Cell Types with Recombinant Lentiviral Particles

The 293T cells (5 × 10^4^ cells/cm^2^) and NSCs (6.5 × 10^4^ cells/cm^2^) were seeded in the appropriate medium and incubated at 37 °C for 16 h. Next, appropriate amounts of RLPs were added to the cells, in which the medium was previously halved. Between 4 and 8 h post-transduction, complete medium was added to the final volume. In most of the experiments, cells were transduced with the same amount of TUs, calculated as explained above. The relative expression of each ubiquibody was always checked in parallel by running an ad hoc western blot, or by immunofluorescence analysis, to control the similar amount of recombinant proteins that were expressed or the similar percentage of cells that were transduced, respectively.

### 2.5. Immunoprecipitation and Immunoblotting 

Immunoprecipitation of flag-tagged proteins was performed by adopting the FLAG^®^ Immunoprecipitation Kit (Sigma-Aldrich), following the manufacturer’s instruction. Briefly, 293T cells (4.8 × 10^4^/cm^2^) were grown in 25 cm^2^ tissue culture flasks and transduced or transfected, as requested. After the appropriate time post-transduction or transfection, cells were lysed with 50 mM Tris HCl, pH 7.4, 150 mM NaCl, 1 mM EDTA, and 1% Triton X-100. Next, columns containing an anti-FLAG^®^-M2 affinity gel were adopted. After formation of the antigen–antibody complex and appropriate washing (washing buffer 10X: 0.5 M Tris HCl, pH 4.0; 1.5 M NaCl), flag-tagged protein dissociation from the immune complex was achieved using the sample buffer (2X: 125 mM Tris HCl, pH 6.8; 4% SDS; 20% (*v*/*v*) glycerol 0.004% (*w*/*v*) bromophenol blue). Whole-cell extract (WCE) was prepared with RIPA buffer 1X (PBS 1X, 1% *v*/*v* NP40, 0.5% *v*/*v* Sodium Deoxycholate, 0.05% *v*/*v* sodium dodecyl sulphate supplemented with protease inhibitors (Roche, Berlin, Germany) 1X) and proteins were quantified by bicinchoninic acid assay (BCA) assay (Thermofisher, Woburn, MA, USA). Next, 30 μg WCE were run on an SDS-PAGE gel, followed by western blot, by adopting either an anti-HA (Boehringer Mannheim, Mannheim, Baden-Württemberg, Germany) or an anti-aS (Abcam, Cambridge, UK) antibody as primary antisera. Target proteins were visualized by chemiluminescence assay (Amersham^™^ ECL^™^ Prime GE Healthcare, Freiburg, Germany) following manufacturer’s instructions.

### 2.6. Analysis of Feline Immunodeficiency Virus (FIV) Gag Ubiquitination

The 293T cells (1.5 × 10^6^) were seeded into 25 cm^2^ tissue culture flasks. A duration of 24 h later, cells were transfected by lipofectamine 2000 reagent (ThermoFisher Scientific, Woburn, MA, USA) with 1.5 μg of a construct containing the Gag/Pol and Rev encoding sequences of the feline immunodeficiency virus (FIV), and p34TF10 strain (NC_001482), bearing a STOP codon in place of amino acid 10 of the protease (PSAPx_5_LLDL/STOP), which we described previously [30]. Furthermore, cells were transfected with 2 μg of pBJ5-HA-Ub, a construct encoding Ub, enriched by an HA tag [31] and 6.5 μg of either pptCMV-NEDD4HA-WT-WPRE or pptCMV-Nac32HECTHA-WT-WPRE. A duration of 48 h post-transfection, the culture supernatants were collected and the cells were lysed in RIPA buffer. Supernatants were clarified by low-speed centrifugation and passaged through 0.45-μm-pore-size filters. Released VLPs were spun for 2 h at 4 °C and 27,000 rpm in a Beckman SW41 rotor. Pelleted VLPs were lysed in RIPA buffer.

For immunoblot analysis, aliquots of lysed VLPs were resolved by SDS-PAGE and electroblotted onto a Hybond-C Extra membrane (Amersham). The membranes were incubated with the appropriate antibody, namely a monoclonal anti-FIV capsid antiserum (anti FIV-p24 Gag, BioRad, Hercules, CA, USA) or a mouse monoclonal anti-HA antibody (Boehringer Mannheim), followed by a peroxidase-conjugated anti-mouse IgG antibody (GE Healthcare). WB with the anti-HA antibody was performed after incubating the membrane blotted with the anti-FIV p24 Gag antibody for 25 min at 50 °C in a stripping buffer (100 mM beta-mercaptoethanol, 2% (*w*/*v*) Sodium Dodecyl Sulfate, 62.5 mM TRIS-Cl pH 6.8). The blots were developed with enhanced chemiluminescence reagents (Amersham), as described [32].

### 2.7. FACS Analysis 

The 293T cells (5 × 10^4^ cells/cm^2^) and NSCs (6.5 × 10^4^ cells/cm^2^) were transduced with RLPs expressing different transgenes, according to the specific experiment. After the appropriate time post-transduction, cells were harvested in cold PBS and pelleted at 1200 rpm for 10 min at 4 °C. Pellets were washed twice in cold PBS, suspended in 500 μL of PBS and analyzed by flow cytometry (FACSCALIBUR; Beckton & Dickinson, Franklin Lakes, NJ, USA).

When requested, NSCs transduced with lentiviral particles expressing Nac32HECT either in the WT or in the C/S form were fixed in paraformaldehyde (4% *v*/*v* in PBS) for 20 min and permeabilized with Triton X-100 (0.1% *v*/*v* in PBS) for 5 min at room temperature. Cells were then incubated with the anti-FOXA2 antibody (Abcam; dilution: 1:200) and with a secondary fluorescein isothiocyanate-conjugated goat anti-rabbit immunoglobulin G antibody (DBA, Milan, Italy; dilution: 1:500).

Cells were pelleted, suspended in PBS, and evaluated by flow cytometry (FACSCalibur; Beckton & Dickinson, Franklin Lakes, NJ, USA). Data analysis was performed by adopting the Flowing software^®^ (Turku Bioscience, Turku, Finland).

### 2.8. Immunofluorescence Analysis

NSCs (6.5 × 10^4^ cells/cm^2^) were grown on poly-ornithine/laminin-coated coverslips and differentiated into dopaminergic progenitors. Cells were fixed in paraformaldehyde (PFA, 4% *v*/*v* in PBS) for 20 min and permeabilized with Triton X-100 (0.1% *v*/*v*) in PBS for 5 min at room temperature. Cells were then incubated with the following primary antibodies diluted 1:200 in BSA (1% *w*/*v* in PBS): anti aS (Abcam), anti-Pax6 (Sigma-Aldrich), anti-Nestin (Abcam), anti-Lmx1A (Abcam), and anti-FOXA2 (Abcam), with a secondary fluorescein isothiocyanate-conjugated goat anti-rabbit immunoglobulin G antibody (DBA; dilution: 1:500) or a secondary Alexa Fluor 568-conjugated goat anti-rabbit (ThermoFisher Scientific; dilution: 1:500). The cells were observed with a confocal microscope (Leica, Buffalo Grove, IL, USA).

### 2.9. Proximity Ligation Assay (PLA)

MN9Dsyn cells (4 × 10^4^ cells/plate) were grown on polyethyleneimine-coated (PEI) glass coverslips in 24-well plates (Nunclon^TM^). The next day, cells were transduced with the appropriate quantities of lentiviral particles expressing the ubiquibodies and, after 24 h, were treated with 1 µg/mL doxycycline (DOX) or left untreated. After 24 h, cells were fixed in PFA (4% *v*/*v* in PBS) for 20 min, permeabilized with Triton X-100 (0.1% *v*/*v* in PBS) for 15 min, and were processed according to the Duolink^®^ manufacturer’s protocol. The following primary antibodies were adopted: anti-human synuclein (syn211, Neomarkers, Portsmouth, NH, USA, Cat.#MS-1572-PABX, 1:1000), anti-HA (Covance, 1:250), and anti-glutamine synthetase (Abcam, Cambridge, UK, Cat.#ab16802, 1:250). Image analysis was performed by adopting the ImageJ software (version number 1.51j, https://imagej.nih.gov/ij/download.html, accessed on 7 February 2017), as previously described [33].

### 2.10. Statistical Analyses

Graphs and statistical comparisons, applying Student’s *t*-test, were performed with the GraphPad Prism version 8.0.0 for Windows (GraphPad Software, San Diego, CA, USA) and statistical differences were determined by *p*-values. For the PLA assay, data were analyzed by using GraphPad Prism 8. One-way ANOVA, followed by Bonferroni post hoc test, was performed to compare the PLA puncta in soma/processes of negative control and samples. Data were expressed as means ± standard deviation of two independent experiments in triplicates, unless otherwise stated. Differences were considered significant when *p* < 0.05.

## 3. Results

### 3.1. Developed Ubiquibodies Are Efficiently Expressed in Human Embryonic 293T Cells

**Figure 1:**
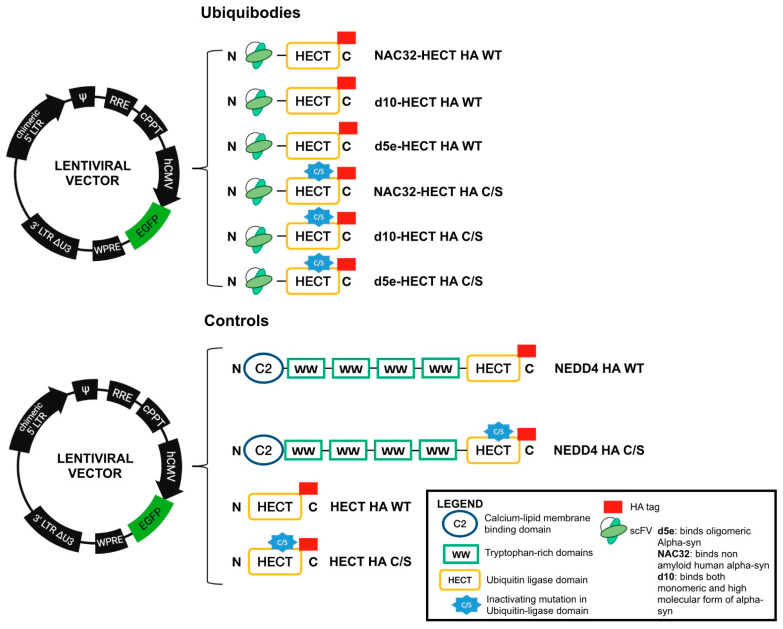
Schematic representation of the developed ubiquibodies, along with the map of the third-generation self-inactivating LV adopted in this study.

The first goal of this study was to generate chimeric ubiquitin (Ub)-ligases, herein named ubiquibodies, consisting of well-characterized single chain variable fragments (scFvs) directed against human aS, fused in frame to the catalytic HECT domain of the NEDD4 Ub-ligase. In particular, three scFv were selected, each with a well-characterized affinity for aS, namely Nac32, d5e, and d10. Selected scFvs were fused to a GSGSG spacer peptide, which provides suitable flexibility to the enzyme [34], followed by the NEDD4 HECT catalytic domain and by an-HA tag. For each ubiquibody, a control bearing a non-functional HECT domain, characterized by the C867S mutation (C/S) within the catalytic domain [18], was also generated. Additional controls were developed—i.e., wild type (WT) and C/S—form of the full length NEDD4-HA and WT and C/S form of the isolated HECT domain. All recombinant proteins were cloned in the context of a third-generation lentiviral vector (LV), as reported in Figure 1.

**Figure 2:**
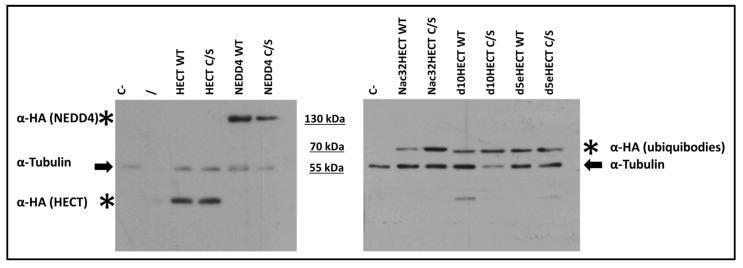
The developed LVs efficiently express ubiquibodies and their controls. All generated LVs (2.5 μg) were transfected in 293T cells. Forty-eight hours post-transfection, a western blot with an HA-specific antibody, recognizing the different ubiquibodies, along with an anti-tubulin antibody, as loading control, was performed. C- stands for untransfected cells; / indicates an empty lane; arrows and asterisks point to tubulin and ubiquibodies/NEDD4/HECT domain, respectively, and as indicated.

Generated constructs were transfected in human embryonic kidney 293T cells. Forty-eight hours post-transfection, cells were lysed and extracted proteins were run in an SDS-gel followed by western blotting. As shown in Figure 2, all the constructs led to the expression of a protein of the expected size, i.e., roughly 100 kDa for NEDD4 WT and NEDD4 C/S; 71 kDa for Nac32HECT WT and Nac32HECT C/S; 68.5 kDa for d10HECT WT and d10HECT C/S; 69 kDa for d5eHECT WT and d5eHECT C/S.

**Figure 3:**
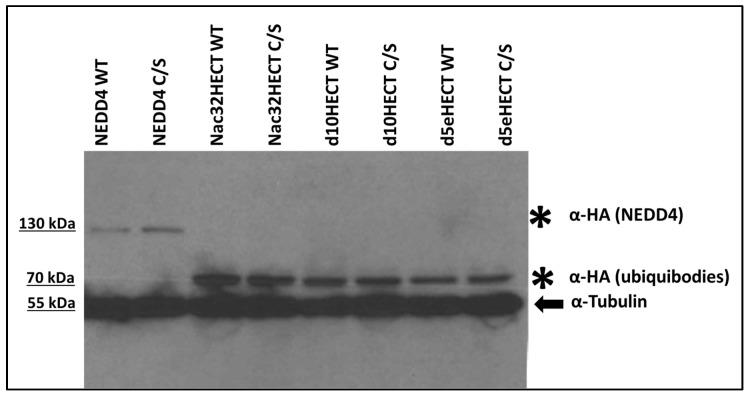
RLPs lead to the expression of the different ubiquibodies in 293T cells. Cells were transduced with RLPs, expressing the following constructs, as indicated: NEDD4 WT; NEDD4 C/S; Nac32HECT WT; Nac32HECT C/S; d10HECT WT; d10HECT C/S; d5eHECT WT; d5eHECT C/S. Forty-eight hours post-transduction, cells were harvested and proteins were run on an SDS-PAGE gel, followed by western blot with an HA antibody, recognizing NEDD4 (~120 kDa bands) as well as the different ubiquibodies (~70 kDa bands), along with an anti-tubulin antibody (~55 kDa bands) as loading control.

Next, recombinant lentiviral particles (RLPs) were produced. To this end, 293T cells were co-transfected with the generated LVs along with the packaging plasmids encoding for the structural and enzymatic proteins needed to assemble viral particles. Forty-eight hours later, cell supernatants were harvested and concentrated. The expression of the different ubiquibodies was evaluated by western blot upon transduction of 293T cells with the generated RLPs. Forty-eight hours post-transduction, 293T cells were lysed and the expression of the different ubiquibodies and of the respective controls was evaluated by HA-specific western blot. Data clearly show that generated RLPs are able to transduce 293T cells and that the transgenes are expressed (Figure 3). 

### 3.2. Generated Ubiquibodies Functionally Interact with aS in a Specific Manner

**Figure 4:**
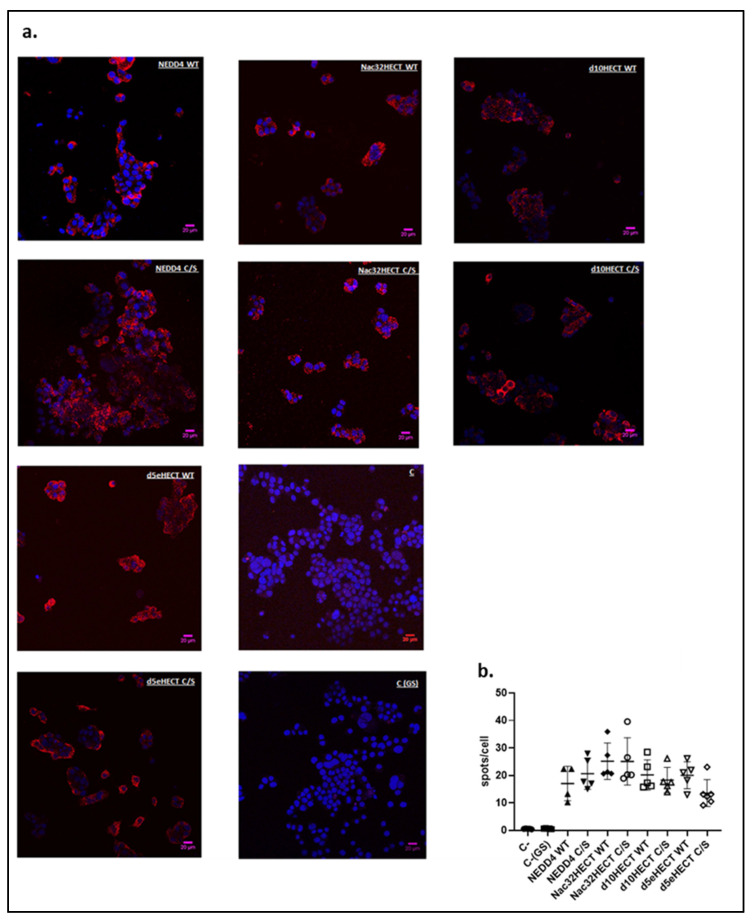
Ubiquibodies specifically interact with aS in murine dopaminergic cells. (**a**) MN9Dsyn cells (4 × 10^4^) were transduced with RLPs expressing similar amount of ubiquibodies and 24 h later, aS expression was induced by treatment with DOX (1 µg/mL). The following day, cells were fixed with 4% *v*/*v* PFA and stained with an anti-HA and an anti-aS antibody, followed by PLA probes minus and plus ligation and amplification. Two negative controls were adopted: (i) cells were incubated with the aS antibody alone (panel C, third line, right panel); (ii) cells were incubated with aS and GS antibodies (C (GS), last line, right panel). Cells were analyzed by confocal microscope. In blue—(DAPI): nuclei; in red—PLA spots. Name of ubiquibodies expressed in the different samples are reported. Shown scale bar corresponds to 20 µm. (**b**) One-way ANOVA (GraphPad Prism version 8.0.0 for Windows, GraphPad Software, Inc, San Diego, CA, USA) statistical analysis was performed by adopting 6 images per each sample. Results are expressed as mean ± SD taken in 6 different regions of the cell culture, from two separate experiments. All the ubiquibody samples have *p* < 0.0001 with respect to the negative control (C-).

As the next step, the ability of generated ubiquibodies to interact with aS in the context of the cellular environment was tested. To this end, an MN9Dsyn murine dopaminergic-like cell line was adopted as it represents a well-established model to study aS biology and therapeutic strategies [25,26]. Indeed, MN9Dsyn cells are engineered with a plasmid that allows the simultaneous expression of aS and GFP upon doxycycline induction. First, different amount of RLPs expressing WT and C/S ubiquibodies were adopted to transduce MN9Dsyn cells. Once transduction conditions led to similar expression of each ubiquibody or control, as shown by HA-specific immunofluorescence analysis (Supplementary Figure S1, HA panels), aS expression was induced in transduced MN9Dsyn cells by doxycycline (DOX) treatment. Twenty-four hours later, cells were fixed, permeabilized, and a proximity ligation assay (PLA) was performed by adopting an anti-aS and an anti-HA (ubiquibodies) antibody. As a negative control, cells were incubated with the aS antibody alone (Figure 4a, panel C). PLA red dots, visible in Figure 4a, clearly indicate that all generated ubiquibodies interact with aS. To further support this conclusion, we set up a nonspecific binding control based on glutamine synthetase (GS) a protein, well expressed in MN9Dsyn cells (Supplementary Figure S1), that does not interact with aS [35]. In this case, upon aS induction, cells were incubated with a (GS) specific antibody along with the aS antibody. The lack of PLA red spots, as shown in panel C(GS) of Figure 4a (last line, right panel), clearly indicates that the assay is correctly working. Importantly, Nac32HECT appears to be overall the best performing ubiquibody in terms of binding to aS. Furthermore, and as expected, the C/S form of each ubiquibody appear to interact more tightly with aS, with respect to the corresponding WT form (Figure 4b).

**Figure 5:**
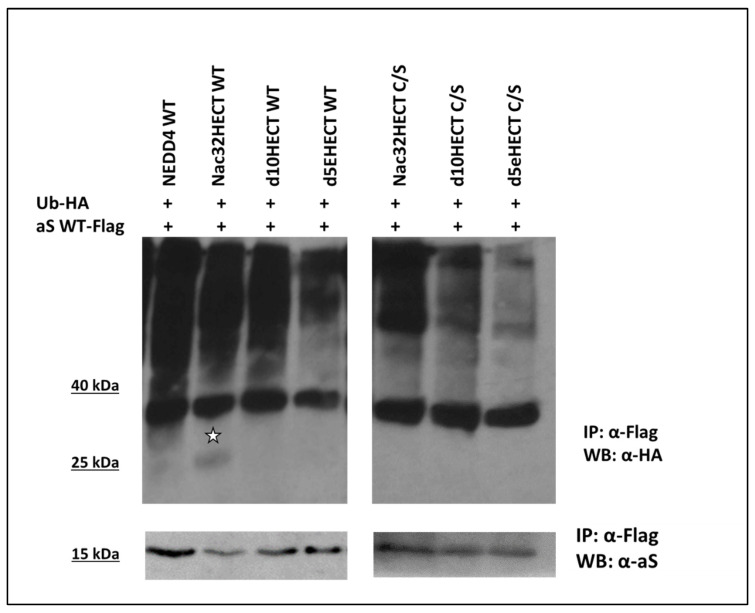
Ubiquibodies expression leads to aS ubiquitination. 293T cells (1.5 × 10^6^) were transfected with LVs expressing ubiquibodies (8 µg) each in the WT or C/S form, along with a plasmid expressing HA-ubiquitin (Ub-HA, 2 µg). Cells were also transfected with LV encoding WT NEDD4 as a control. Twenty-four hours later, cells were transduced with RLPs expressing aS WT fused in frame with a flag tag (aS WT-Flag). In order to recover aS from the cell lysates, 48 h post-transduction, cells were lysed and proteins were subjected to a flag-immunoprecipitation, followed by an anti-HA, to recognize ubiquitinated forms of aS (**upper panel**), or an anti-aS western blot (**lower panel**) to control for aS recovery from the cell lysates. Star points to a band corresponding in size to mono-ubiquitinated aS. Results were replicated 3 times in independent experiments with similar results.

Having demonstrated a binding between aS and ubiquibodies, we tested the ability of WT ubiquibodies to ubiquitinate aS. To this end, 293T cells were transfected with the appropriate LVs, along with a construct expressing Ub enriched with an HA tag (Ub-HA). Twenty-four hours later, cells were transduced with RLPs expressing WT aS enriched with a 3′ end flag epitope (aS WT-Flag). Forty-eight hours post-transduction, cells were harvested and lysed. Next, aS was immunoprecipitated from protein lysates by adopting a flag-specific antibody, followed by an HA western blot (Figure 5).

As shown in Figure 5, in the presence of full length NEDD4 (lane 1) a smear above aS appears, as expected in the case of protein ubiquitination. A similar result is obtained in the case of Nac32HECT as well as of d10HECT expression (lanes 2 and 3), while the smear is less evident in the presence of d5eHECT (lane 4) and of the C/S versions of the different ubiquibodies (lane 5–7). Of note, when Nac32HECT is expressed, a band corresponding in size to mono-ubiquitinated aS (star, lane 2) is appreciable. Overall, these data indicate that ubiquibodies and in particular Nac32HECT leads to aS ubiquitination.

**Figure 6:**
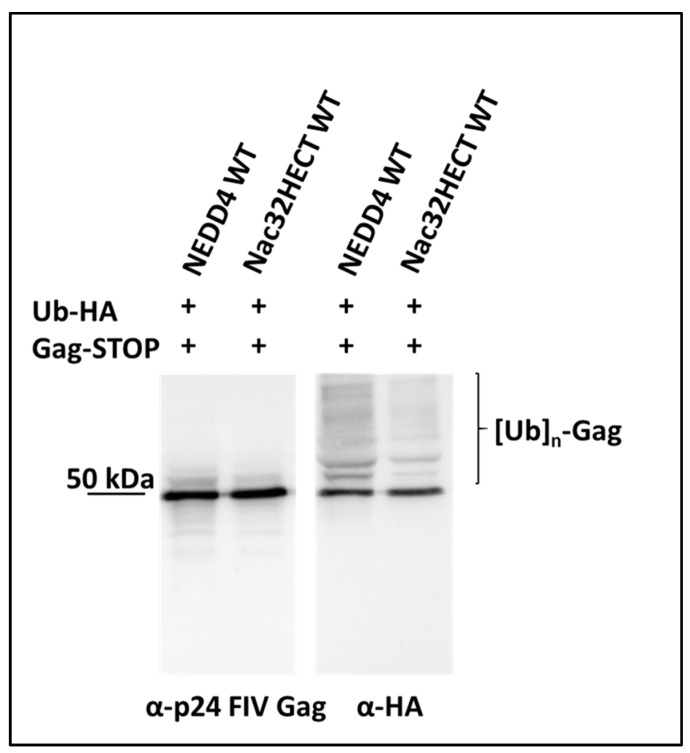
Nac32HECT expression does not affect ubiquitination levels of FIV Gag, a physiological target of NEDD4-like Ub-ligases. 293T cells were co-transfected with constructs expressing FIV Gag-STOP (1.5 µg) and HA-tagged Ub (Ub-HA, 2µg), along with LVs encoding either NEDD4 or Nac32HECT (6.5 µg), as indicated. Purified VLPs were subjected to SDS-PAGE, followed by western blotting analysis, as reported. [Ub]_n_-Gag stands for ubiquitinated forms of FIV Gag. Results were replicated 3 times in independent experiments with similar results.

We and other research groups have previously shown that Ub-ligases belonging to the NEDD4 family are able to ubiquitinate the retroviral structural protein Gag, influencing particle release from infected cells [32,36,37,38,39,40,41,42,43,44,45,46,47]. In order to analyze the specificity of Nac32HECT recognition of and binding to aS, we transfected 293T cells with the LVs expressing either NEDD4 or Nac32HECT, along with constructs encoding Ub-HA and the unprocessed form of the feline immunodeficiency virus (FIV) Gag (Gag-STOP). Forty-eight hours post transfection, virus-like particles (VLPs) were harvested from the cell supernatants and loaded on an SDS-PAGE followed by WB specific for FIV Gag as well as for the HA tag. Data reported in Figure 6 show that while, as expected, NEDD4 expression increases the basal levels of FIV Gag ubiquitination, Nac32HECT does not. This finding, along with the results reported in Figure 5, demonstrate that Nac32HECT lacks the ability to functional interact with a physiological target of NEDD4, whilst being able to ubiquitinate aS.

### 3.3. Nac32HECT Ubiquibody Affects aS Intracellular Levels

**Figure 7:**
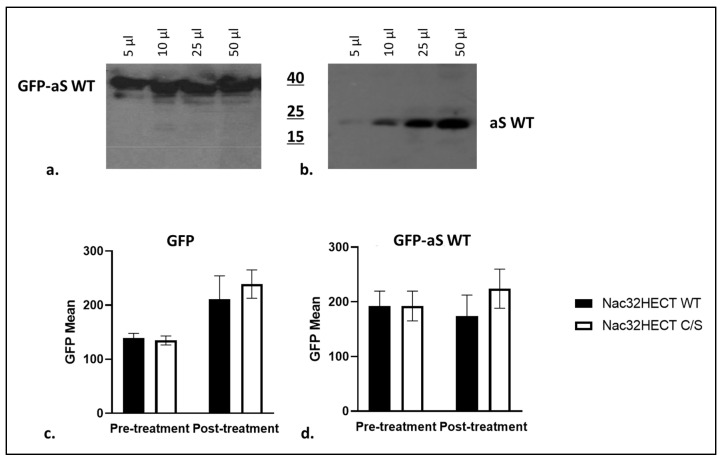
WT Nac32HECT reduces aS intracellular levels. Cells were transduced with RLPs expressing either GFP- aS WT (**panel a**) or aS WT (p**anel b**) to assess by western blot the presence of a single band of the expected size in the sample harvested from cells expressing the fusion protein. Next, 293T cells were transduced with RLPs expressing either GFP alone (**panel c**) or GFP-aS WT (**panel d**) and, the day after, with RLPs expressing comparable amounts of WT or C/S Nac32HECT (black and white, respectively). Cells were harvested and green fluorescence derived from GFP was measured by cytofluorimetric analysis, before (pre-treatment) and after (post-treatment) treatment with ubiquibodies. Specifically, the geometric mean of green fluorescence (GFP Mean) was measured as reported in the y axis. 10,000 events were analyzed. Results are expressed as mean ± SD taken from three independent experiments of the cell culture.

Based on the PLA and ubiquitination results, we selected Nac32HECT for further investigation of its effects on aS. First, we generated a lentiviral vector expressing wild-type aS, fused in frame with GFP (GFP-WT aS). RLPs carrying this vector, once transduced in 293T cells, led to the expression of a fusion protein of the expected size, running higher than aS alone (Figure 7a,b, respectively). Next, 293T cells were transduced with RLPs expressing either GFP-aS WT or GFP alone at the multiplicity of infection (MOI) of 2 TUs/cell. Twenty-four hours later, part of the cells were collected and underwent FACS analysis for GFP expression. The remaining cells were transduced with RLPs, expressing either WT or C/S Nac32HECT at the MOI of 5 TUs/cell. Forty-eight hours post-transduction, the intensity of the GFP signal was measured by FACS analysis (Figure 7c,d). 

In cells expressing GFP-WT aS, the geometric mean of GFP, that represents the signal intensity, is reduced (Figure 7d black bar, post-treatment columns). By contrast, and as expected due to cell growth, this value increases with respect to the pre-treatment samples when Nac32HECT C/S is adopted (Figure 7d white bar, post-treatment columns). Importantly, in the case of GFP alone, neither WT nor C/S Nac32HECT expression led to a reduction of the geometric mean post-treatment (Figure 7c). Although the observed reduction is not statistically significant (*p* = 0.2463), overall, these data suggest that Nac32HECT expression affects aS intracellular amount in a specific manner. 

Further supporting this conclusion, we adopted a more physiological setting represented by the neuroblastoma SH-SY5Y cell line, which expresses dopaminergic markers, and it is widely adopted to study PD pathogenesis/treatment. Cells were co-transfected with a construct expressing aS along with LVs encoding Nac32HECT both in the WT and C/S version. Twenty-four hours later, cells were fixed, incubated with an aS specific antibody, and analyzed by confocal microscopy in order to quantify the aS signal intensity. 

**Figure 8:**
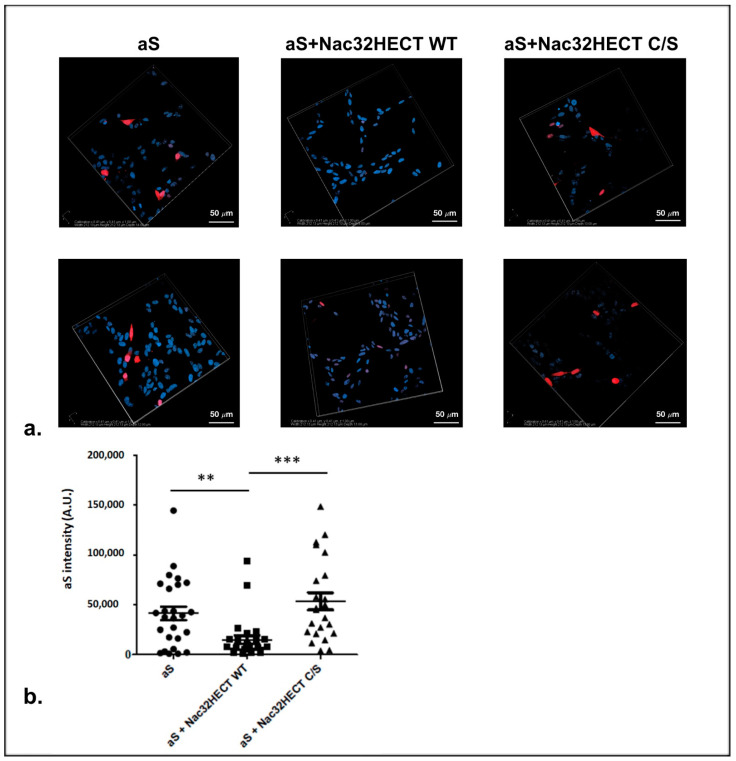
Nac32HECT overexpression affects aS intracellular amounts. Two µg of a plasmid expressing aS (pHM6-alphasynuclein-WT) were transfected in the neuroblastoma SH-SY5Y cell line alone or in combination with 2 µg of LVs encoding for either Nac32HECT WT or Nac32HECT C/S. Twenty-four hours later, cells were fixed with PFA (4% *v*/*v*), permeabilized with Triton (0.5% *v*/*v*) and incubated with an anti-aS (Abcam) antibody, followed by an anti-rabbit rhodamine secondary antibody. Nuclei were marked with DRAQ5™ (ThermoFisher Scientific). The glasses were mounted in glycerol-PBS media and analyzed by confocal microscopy, by maintaining the same laser setting for all the acquisitions. Confocal imagines are reported in (**panel a**), while (**panel b**) shows the statistical analysis performed by adopting five z-stack images per each sample from a single experiment (*t* test, *** *p* < 0.0001 and ** *p* < 0.001). Scale bar in panels “a” corresponds to 50 µm.

As shown in Figure 8, Nac32HECT expression led to a significant reduction of aS specific signal, while Nac32HECT C/S expression did not.

Finally, we tested the effect of Nac32HECT on endogenous aS intracellular amount over time. To this end, SH-SY5Y cells were transduced with RLPs expressing Nac32HECT, either WT or C/S, at a MOI of 5 TU/cell. Twenty-four hours later, cells were incubated in fresh medium containing 20 μg/mL of cycloheximide (Chx) to block protein synthesis, as previously reported [17]. Cells were harvested at different time points post-treatment and endogenous aS was revealed by western blot. The housekeeping glyceraldehyde-3-phosphate dehydrogenase (GAPDH) protein was adopted as loading control and to normalize the intracellular amount of aS over time.

**Figure 9:**
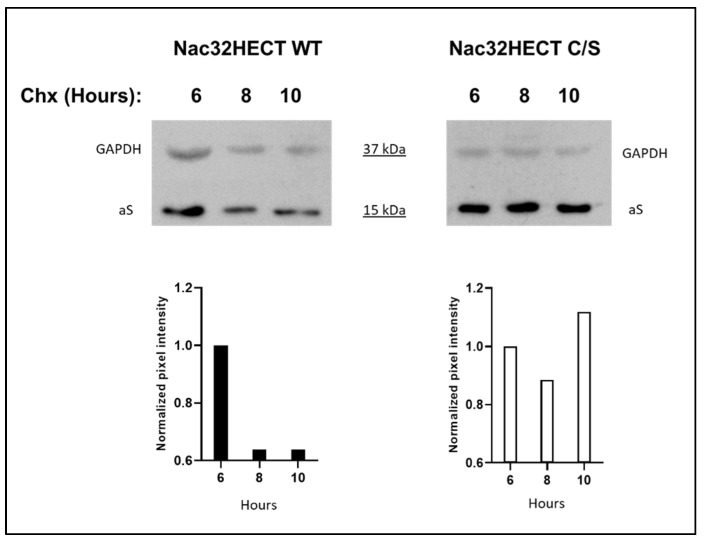
Intracellular amounts of endogenous aS are affected by Nac32HECT expression in neuroblastoma-derived cells. SH-SY5Y cells were transduced with RLPs expressing Nac32HECT WT or Nac32HECT C/S. Twenty-four hours later, cells were incubated in fresh medium containing 20 μg/mL cycloheximide (Chx), harvested at 6, 8, and 10 h post-treatment, as indicated and lysed. A western blot was performed by adopting an anti-GAPDH and an anti-aS antibody. Values reported in the graphs below each western blot represent the ratio of the pixel intensity of the target protein band to that of the respective loading control, with the ratio calculated for 6 h post-treatment set to 1. Quantification of band intensities was conducted by densitometry analysis (ImageJ software; version number 1.51j).

Results reported in Figure 9 show that while the expression of Nac32HECT reduces the amount of endogenous aS over time (up to 10 h post-treatment), the expression of its catalytically inactive form does not.

Overall, our findings indicate that Nac32HECT not only binds and ubiquitinates aS, but also leads to its degradation.

### 3.4. Nac32HECT Rescues the Ability of NSCs to Differentiate into Dopaminergic Progenitors

It has been previously shown that aS overexpression affects the ability of neural stem cells (NSC) to differentiate into dopaminergic neurons (DNs) [48]. To test whether Nac32HECT effects on aS have biological consequences, hiPSCs derived from PD patients either bearing *SNCA* triplication (#49) or carrying A53T aS (#52) were differentiated into NSCs (Supplementary Figure S2 and Supplementary Table S1). In parallel, as a control, hiPSCs derived from a healthy control (UNIPDi007-A) were also adopted and differentiated in NSCs (Supplementary Figures S2 and S3a, and Supplementary Table S1). UNIPDi007-A-derived NSCs were either transduced with RLPs expressing WT aS. Twenty-four hours later, all NSCs samples were differentiated in dopaminergic progenitors (DPs), by incubation in a specific media supplemented with N2, Sonic Hedgehog (SHH), and Fibroblast Growth Factor 8 (FGF8). One week later, after assessing differentiation (Supplementary Figure S3b), cell viability was evaluated by propidium iodine (PI) staining, followed by FACS analysis. Data reported in Supplementary Figure S4 show that *SNCA* triplication or mutation results in a significant increase of cell mortality with respect to control cells. 

**Figure 10:**
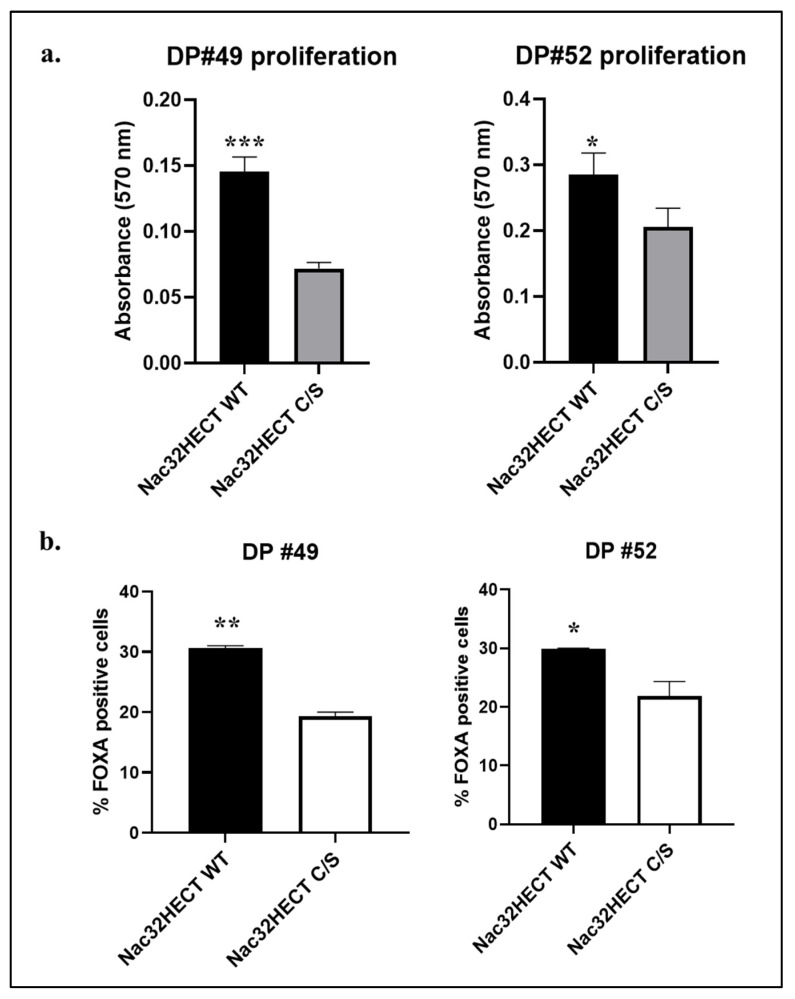
WT Nac32HECT ubiquibody rescues PD NCS differentiation into DPs. NSCs #49 and #52 were transduced with RLPs expressing similar amounts of WT and C/S Nac32HECT ubiquibodies and cells were differentiated into DPs. Next, (**a**) a proliferation assay was performed by adopting the commercial kit CyQUANT™ NF Cell Proliferation Assay (ThermoFisher Scientific), along with (**b**) an evaluation of FOXA2 positive cells by FACS analysis. Statistical differences were determined by *p*-values of * *p* < 0.0332; ** *p* < 0.002; *** *p* < 0.0002.

Having confirmed the effects of aS alteration in our experimental setting, we tested whether Nac32HECT expression affected NSCs biological properties during their differentiation into DPs. In this set of experiments, we focused our attention on PD patient-derived cells, as those represent a more relevant model of aS accumulation. Thus, NSCs bearing *SNCA* triplication (NSC #49) or carrying A53T aS (NSC #52), transduced with RLPs expressing similar amounts of WT or C/S Nac32HECT, were treated as reported above. Six days post-treatment, cell proliferation was assayed (Figure 10a), along with the expression of FOXA2, a well-established DP marker (Figure 10b). Data indicate that cells proliferate better in the presence of WT Nac32HECT and display significantly higher percentages of FOXA2 positivity.

Overall, these data suggest that WT Nac32HECT, by interfering with aS intracellular amounts, partially rescues PD patient-derived NSC’s ability to differentiate in DPs. 

## 4. Discussion

The main pathological features of Parkinson’s disease (PD) are the death of dopaminergic neurons and the diffuse accumulation of alpha-synuclein (aS) aggregates in neurons [5]. The NEDD4 E3 ubiquitin (Ub)-ligase promotes aS degradation by the endosomal/lysosomal route. Interestingly, NEDD4 (i) is overexpressed in the brain regions presenting Lewy pathology and a single nucleotide polymorphism in its coding sequence is associated with a greater risk of PD onset [17]; (ii) is protective against human aS toxicity in evolutionary distant models [18]; (iii) a small molecule able to activate NEDD4 functions, has an effect on aS toxicity [19]. The aim of our study was to exploit chimeric Ub-ligases, named ubiquibodies, specifically targeting aS, to prove the neuroprotective role of aS degradation pathway. Portnoff and coworkers had previously demonstrated the feasibility of generating chimeric Ub-ligases directed against a single specific target [20]. In particular, those authors successfully developed a chimera, based on the enzymatic domain of the carboxyl terminus of Hsc70-interacting protein (CHIP) Ub-ligase, fused to a scFv, directed towards a protein of interest. At the same time, the engineered CHIP loses the ability to recognize its physiological targets [20]. Furthermore, Göttlinger and collaborators showed that NEDD4 activity can be directed to the Gag structural protein of the human immunodeficiency virus by fusing the E3 catalytic HECT domain to heterologous proteins known to bind Gag [21].

Based on these findings, we developed an array of lentiviral vectors (LVs) encoding chimeric NEDD4 Ub ligases (ubiquibodies) specifically targeting aS. To this end, we selected three previously well-characterized scFvs, as follows: Nac32, directed against the non-amyloid component (NAC) domain of human aS [22]; d5e, which exclusively binds the oligomeric form of aS [23]; and d10, which is pan-specific [24]. These scFvs were fused to a GSGSG spacer peptide, which provides suitable flexibility to the enzyme [34], followed by the NEDD4 HECT catalytic domain. 

The choice of LVs is justified by the fact that these vectors have shown high efficiency in in vivo gene transfer and a stable and lasting expression of transgenes in multiple target tissues, including in non-dividing cells, like neurons [49]. Furthermore, last generation LVs are currently in several clinical, trials also at advanced stages, for the treatment of genetic disorders [50], thus supporting their safety and efficacy. Finally, we selected LVs over adeno-associated virus (AAV)-based vectors, which are extremely efficient in transducing neurons, based on their superior ability to accommodate large and complex transgenes within their genome [49]. Finally, we adopted the vesicular stomatitis virus (VSV) G, as envelope glycoprotein, as it allows the transduction of a wide range of cell types, including neurons [51]. We were able to show that the generated recombinant lentiviral particles (RLPs) efficiently transduce human embryonic kidney 293T cells, leading to the expression of all the ubiquibodies, thus indicating that the developed vectors are correctly incorporated into the particles.

It has been previously reported that overexpression of WT aS under the transcriptional control of a strong promoter (e.g., the HCMV promoter), either alone or fused in frame with the reporter, enhanced green fluorescent protein (EGFP) leads to aS accumulation in eukaryotic cells resembling what is found in dopaminergic neurons of PD patients [52]. Furthermore, it has been shown that aS bearing the A53T mutation, that is found in an autosomal dominant form of PD [52,53,54], is particularly prone to form fibrils when expressed in vitro in cell lines of different origin [52]. Indeed, overexpression of WT or A53T aS alone or fused in frame with GFP in cells as the 293T cells is widely recognized and adopted as in vitro model for studying strategies to interfere with aS accumulation [52]. With this in mind, we generated LVs expressing WT and A53T aS either alone or fused in frame to GFP under the transcriptional control of the HCMV promoter. Next, we set up to investigate whether ubiquibodies are able (i) to interact with aS; (ii) to lead to aS ubiquitination; (iii) to affect aS intracellular amount. In particular, we were interested in comparing the performance of the three different scFvs, considering their ability to bind different forms and domains of aS (see above). We were able to show that all the developed ubiquibodies bind to and ubiquitinate aS. However, Nac32HECT is overall the best performing recombinant Ub-ligase both in terms of binding and catalytic activity towards aS. Specifically, by adopting a proximity ligation assay (PLA), we demonstrated that Nac32HECT efficiently interacts with aS in the cellular context. Of note, PLA was performed in immortalized murine dopaminergic-like cells, the MN9Dsyn cell line, that are a well-recognized model to study the effects of aS accumulation [25,26]. Furthermore, in this assay the ability of aS to interact with an unrelated protein—i.e., the glutamine synthetase protein (GS)—was checked, thus supporting ubiquibody–aS interaction in the performed assay. Furthermore, in the case of Nac32HECT, we were able to show that while this ubiquibody efficiently ubiquitinates aS, it does not functionally interact with a physiological target of the NEDD4 like Ub-ligases, the FIV structural protein Gag [36]. This result, along with the finding that Nac32HECT was able to reduce aS intracellular amounts while it did not affect an unrelated protein, supports the specificity of Nac32HECT binding and functional interaction with aS. Finally, Nac32HECT was also capable of decreasing endogenous aS levels in a neuroblastoma-derived cell line.

To move to an even more relevant in vitro system, we then adopted dopaminergic neurons, derived from human induced pluripotent stem cells (hiPSCs), obtained from PD patients. The reason for selecting this model is that, as already mentioned, dopaminergic neurons are the cell type in the CNS most affected by aS accumulation in PD patients [52]. Furthermore, the idea of deriving neurons from hiPSCs originated not only from the feasibility of this technique [55] but also from the fact that the process leading from hiPSCs to dopaminergic progenitors (DPs) constitutes, per se, an excellent model to biologically validate strategies aimed at interfering with aS toxicity. Indeed, it has been previously demonstrated by Oliveira and collaborators that high levels of aS have a negative impact on the differentiation of hiPSCs into neural stem cells (NSCs) and into DPs [48]. Importantly, when the expression of aS was silenced by using small interfering RNAs in hiPSCs, the differentiation or maturation capacity of hiPSCs correlated with the degree of silencing [48]. Thus, we decided to adopt the same experimental setting to analyze whether Nac32HECT, by interfering with aS intracellular levels, could rescue the ability of PD patients derived hiPSCs to differentiate into dopaminergic neurons. First, we established a protocol to differentiate hiPSCs into dopaminergic neurons by modifying the method described by Borg and coworkers [55]. Next, we selected two hiPSC lines, one deriving from a PD patient carrying a triplication of the aS encoding gene and the other one obtained from a PD patient characterized by A53T aS. We then confirmed that, as reported by Oliveira and coworkers [48], aS overexpression affects NSC viability, thus validating our experimental conditions. Next, we were able to show that Nac32HECT can partially rescue the viability and ability of NSCs derived from PD patients to differentiate into DPs. Overall, our data suggest that Nac32HECT interferes with aS neurotoxicity, most likely through the binding and degradation of its aggregated forms. Importantly, the scFv Nac32 alone is able to inhibit aS aggregation and rescue its cytotoxicity in cellular models [22].

In conclusion, Nac32HECT represents a suitable tool to interfere with aS intracellular amounts and might be useful not only to further clarify the role of aS in PD pathogenesis, but also as an innovative therapeutic strategy. Previous attempts to develop gene therapy approaches against PD aimed at interfering with aS expression (for instance, by RNA interference) failed, since aS displays physiological functions that need to be preserved [56]. As already mentioned, scFv Nac32 preferentially binds to aggregated aS [22]. If this feature is retained in the context of Nac32HECT, the expression of this ubiquibody should result in a specific post-translational silencing of non-physiological forms of aS, thus overcoming the issues of the siRNA-based strategy. As this aspect is crucial, its validation in suitable experimental models is mandatory and will represent the next step of this work.

## Data Availability

All data are available in the manuscript and the Supplementary Materials. Detailed datasets used and analyzed during the current study, as well as the original WB films and agarose gels are available from the corresponding author on request.

## Supplementary Materials

The following are available online at https://www.mdpi.com/article/10.3390/cells10113256/s1, Figure S1: Ubiquibodies are expressed in murine a dopaminergic cell line, Figure S2: Upon differentiation hiPSCs express markers specific for NSCs, Supplementary Table S1: Oligonucleotides adopted to assess hiPSCs differentiation into NSCs by RT-PCR, Figure S3: Analysis of differentiation from NSC to DPs by immunostaining, Figure S4: Alpha-synuclein overexpression or mutation results in a significant increase of DP mortality.
