# Supplementary material for: Lentiviral Vectors Expressing Chimeric NEDD4 Ubiquitin Ligases: An Innovative Approach for Interfering with Alpha-Synuclein Accumulation
Source: Cells. 2021 Nov 21;10(11):3256. doi: 10.3390/cells10113256 (PMC8624294; doi:10.3390/cells10113256)
Supplement: Supplementary file 1 [file cells-10-03256-s001.zip › cells-1413000-supplementary.pdf]

Supplementary Figures

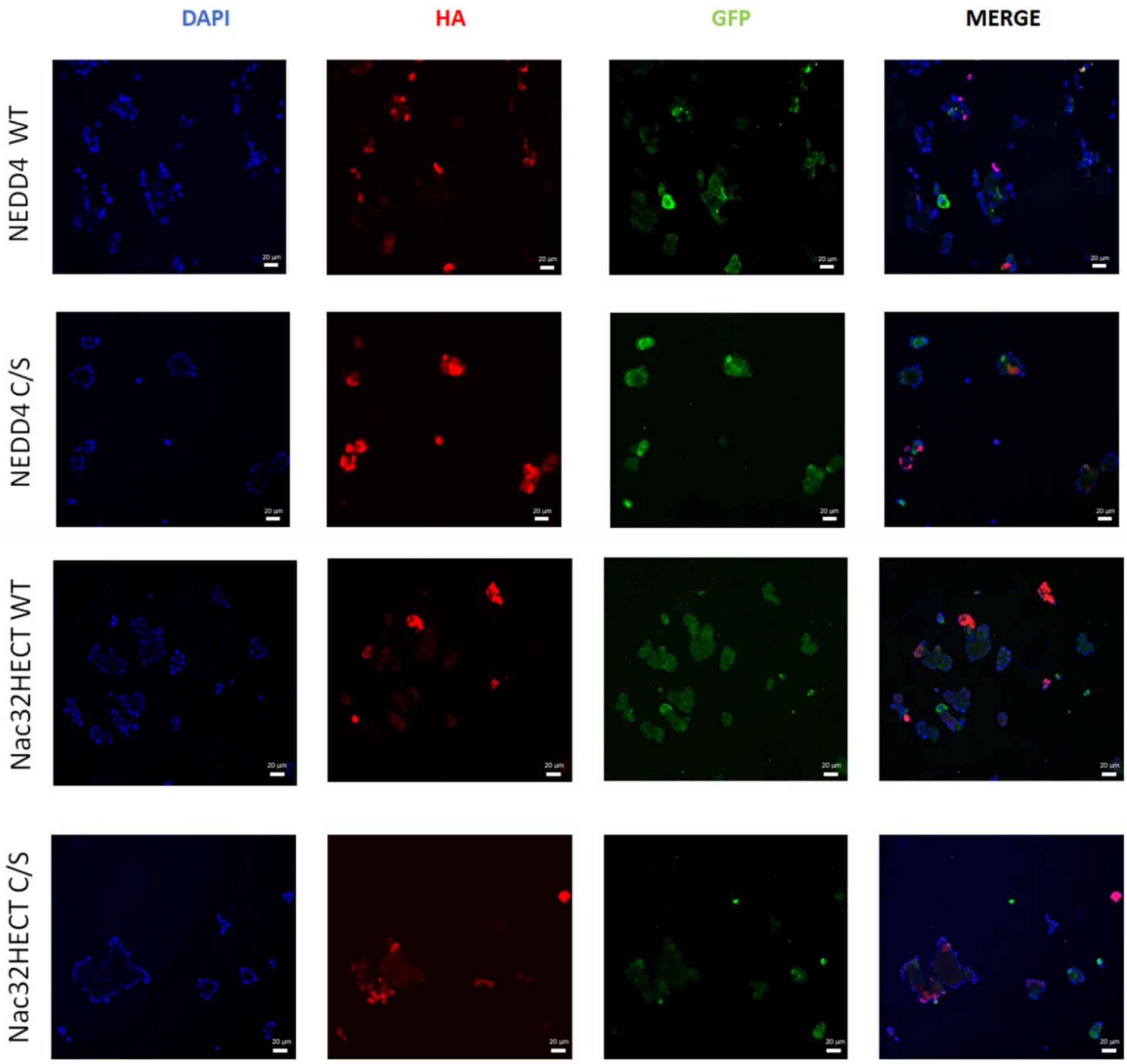

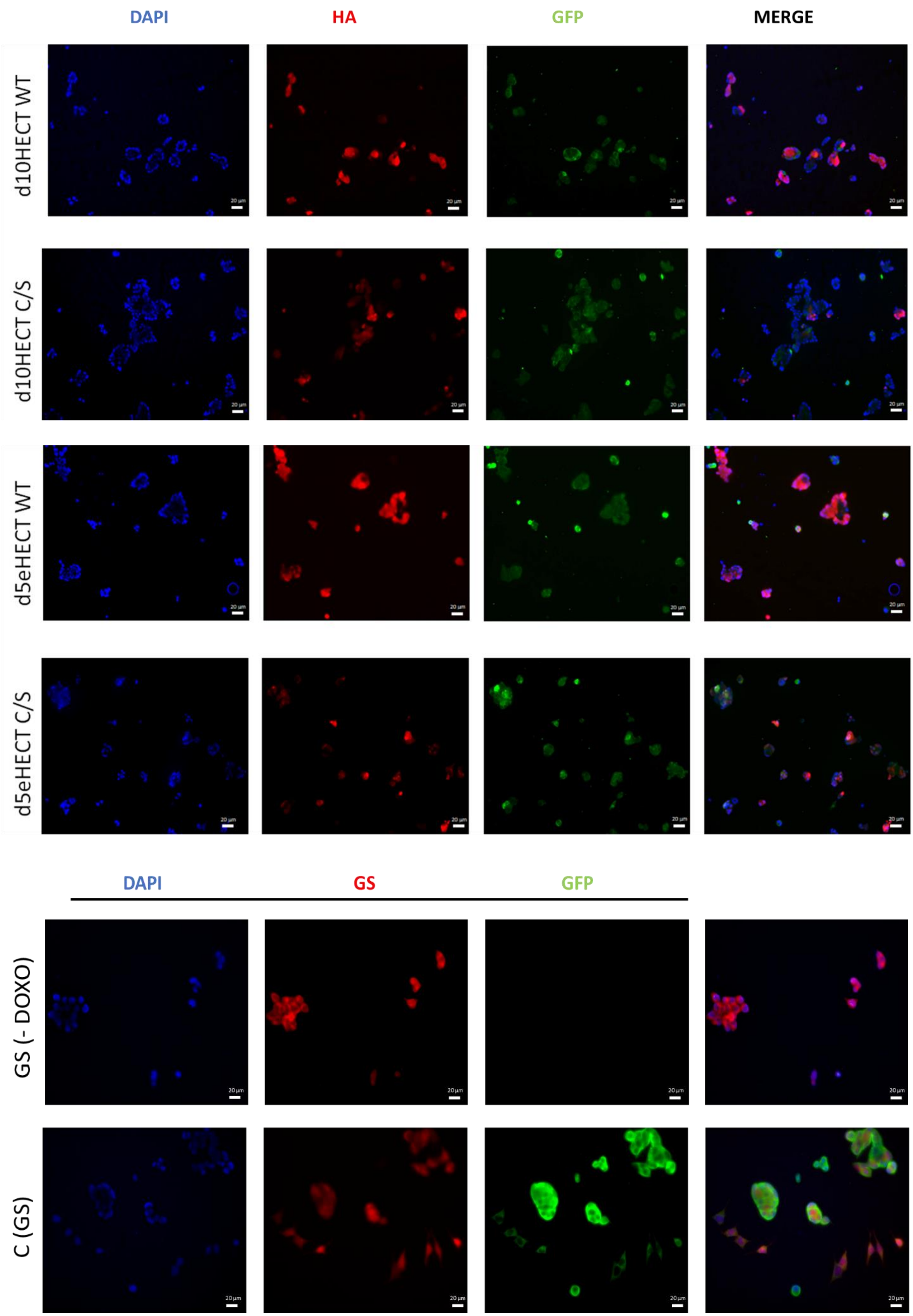

Figure S1. Ubiquibodies are expressed in murine a dopaminergic cell line. MN9Dsyn cells ($4x10^4$) were transduced with RLPs expressing either NEDD4 or the ubiquibodies (WT and C/S forms). Twenty-four hours later the expression of aS

and GFP was induced by doxycycline (DOX) treatment. The following day, cells were fixed by PFA 4% (v/v) and stained with anti-HA. As a control an anti-GS was adopted in cells either DOX treated or untreated. In blue: dapi; in red: HA or GS; in green: GFP. The name of each ubiquibody is reported on the left side. Scale bar corresponds to 20 µm. Cells were observed with a confocal microscope.

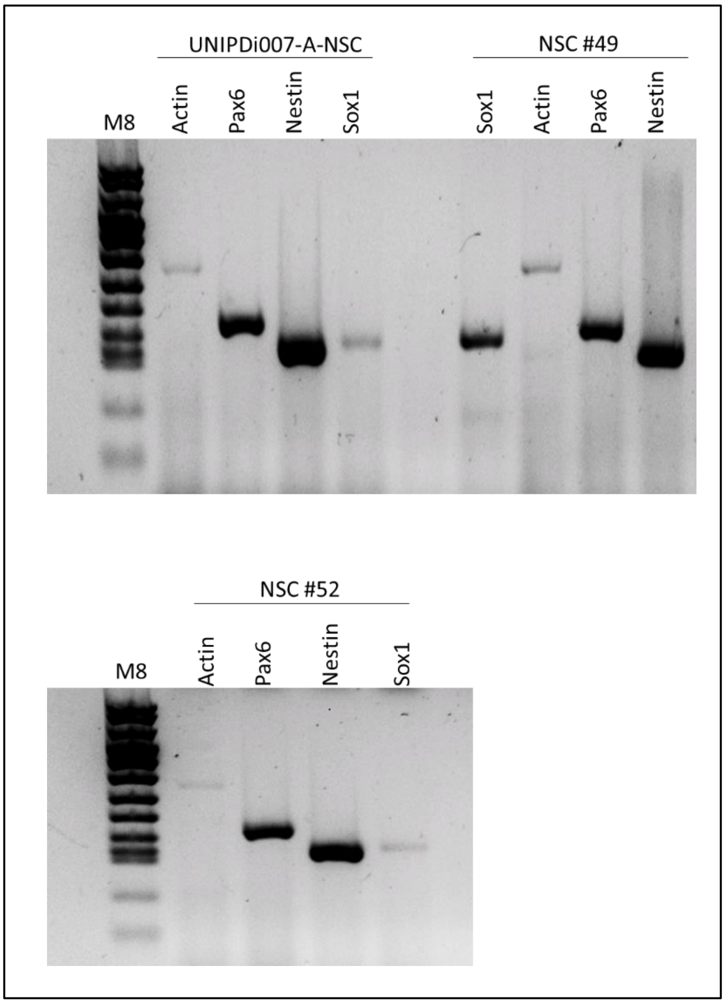

Figure S2. Upon differentiation, hiPSCs express markers specific for NSCs. The expression of different marker of NSCs was evaluated by RNA reverse transcription (RT) followed by endpoint PCR starting from total RNA extracted from the indicated lines of NSCs. Specifically, Pax6, Nestin and Sox1 as well as Actin as a control were checked as reported. M8: DNA Molecular Weight Marker VIII (SIGMA) Size range**:** 19 to 1114 bp.

**Name**	**Sequence 5’-3’**	**RefSeq**	**Ref**
βACT Fw	CCATCGAGCA CGGCA	NM_001101.4	In house designed
βACT Rv	CAGGATGGCATGGGG	NM_001101.4	In house designed
Nestin Fw	CTCCAGCTTTCAGGACCCCAA	NM_006617.1	[55]
Nestin Rv	GTCTCAAGGGTAGCAGGCAA	NM_006617.1	[55]
Pax6 Fw	TCTTTGCTTGGGAAATCCG	NM_000280.4	[56]
Pax Rv	CTGCCCGTTCAACATCCTTAG	NM_000280.4	[56]

Sox1 Fw	CAACCAGGACCGGGTCAAAC	NM_005986.2	[55]
Sox1 Rv	CCTCGGACATGACCTTCCAC	NM_005986.2	[55]

Supplementary Table S1. Oligonucleotides employed to assess hiPSCs differentiation into NSCs by RT-PCR, as reported in supplementary figure S2.

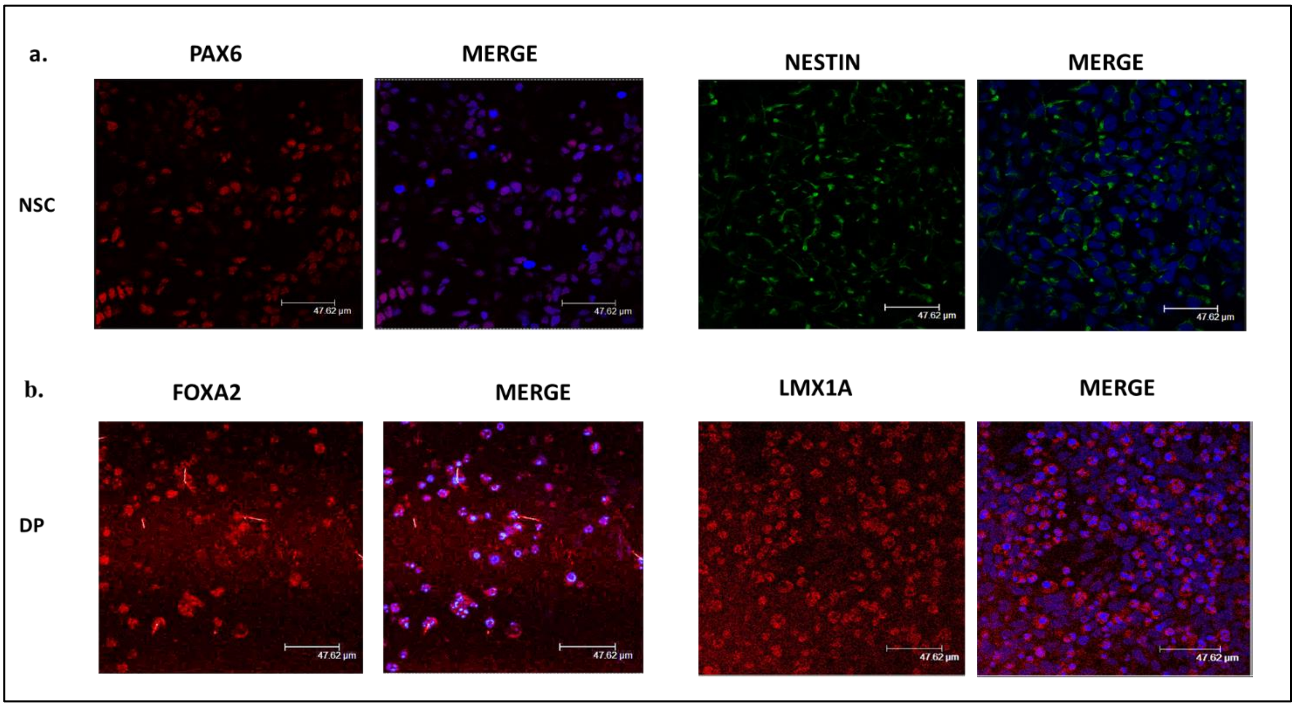

Figure S3. Analysis of differentiation from NSC to DPs by immunostaining. (a) PAX6 and NESTIN expression in NSCs; (b) FOXA2 and LMX1A expression in DPs. Images of the UNIPDi007-A cell line are displayed.

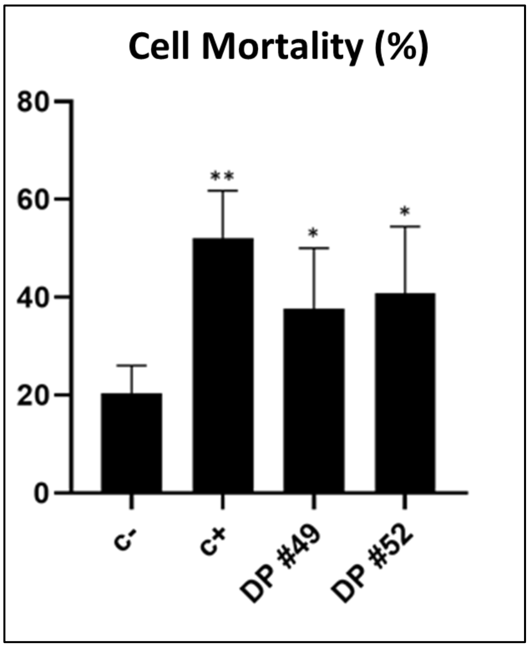

Figure S4. Alpha-synuclein overexpression or mutation results in a significant increase of DP mortality. NSC from healthy donor were transduced with RLPs expressing aS fused in frame with EGFP (c+) and after one week from differentiation, all four lines were observed at the fluorescence microscopy. Propidium Iodide staining was adopted to evaluate the number of dead cells during differentiation and the results were reported in the graph. Statistical differences were determinate by comparing all samples to the untransduced cells from the healthy donor (c-), by P-values of *$P < 0.05$; **$P < 0.005$.
